# Awareness and Attitudes of University Students in Bangladesh Toward Cancer: Cross-Sectional Study

**DOI:** 10.2196/75651

**Published:** 2025-11-13

**Authors:** Maliha Tabassum, Nafisa Farhin, Afsana Afrose, Mahfuza Moin Surovy, Rubaiya Tasnim, Most. Humayra Affia Heaven, Jannatun Noor, Munima Haque

**Affiliations:** 1 Biotechnology Program, Department of Mathematics and Natural Sciences, BRAC University, Kha 224, Bir Uttam Rafiqul Islam Ave, Dhaka, 1212, Bangladesh, 02222-264051; 2 Department of Computer Science and Engineering, BRAC University, Dhaka, Bangladesh; 3 Department of Computer Science and Engineering, United International University, Dhaka, Bangladesh

**Keywords:** cancer awareness, university students, Bangladesh, cancer screening, aging

## Abstract

**Background:**

Early detection and awareness are critical in reducing the burden of cancer. However, a significant proportion of university students in Bangladesh remains inadequately informed about cancer risks and preventive measures.

**Objective:**

This study aimed to assess knowledge gaps and evaluate the attitudes of Bangladeshi university students toward cancer, its prevention, risk factors, and care for affected individuals.

**Methods:**

A descriptive, cross-sectional survey was conducted among 530 university students aged 20 to 35 years across Bangladesh. Data were collected using an ethically approved, structured internet-based questionnaire between December 2022 and March 2024. The questionnaire assessed sociodemographics, cancer knowledge, awareness of risk factors, personal or familial cancer experiences, and attitudes toward cancer care and policy. Descriptive statistics and chi-square tests were used to analyze categorical data, with a significance threshold of *P*<.05.

**Results:**

Most participants were aged 21–25 years (406/530, 76.6%) and female (320/530, 60.4%), with the majority enrolled in undergraduate programs (82.8%, 439/530). While 60.8% (322/530) considered themselves somewhat knowledgeable about cancer, only 11.9% (63/530) were very knowledgeable, and 93.6% (496/530) had never undergone any cancer screening. Despite this, 74.3% (394/530) had personal or familial exposure to cancer, with carcinoma reported by 52.8% (280/530) of those affected. Awareness of established risk factors was inconsistent—smoking (90.9%, 482/530) and radiation (86.6%, 459/530) were widely recognized, but only 38.9% (206/530) acknowledged aging, 35.3% (187/530) obesity, and 29.2% (155/530) infectious agents as risk factors. Reproductive factors were least recognized, with just 10.2% (54/530) identifying having more children as a risk factor. Gender differences were significant in cancer-related attitudes. For example, 51.5% (273/530) of female participants versus 33.4% (177/530) of male participants felt comfortable around patients with cancer (*P*=.01), and 57.2% (303/530) of female participants versus 35.8% (190/530) of male participants supported increased government funding for cancer care (*P*=.03). Furthermore, 55.1% (292/530) of females and 35.5% (188/530) of males stressed the need for enhanced cancer awareness programs (*P*=.05). Only 6.4% (34/530) of all participants reported undergoing any form of cancer screening, highlighting a disconnect between awareness and preventive action.

**Conclusions:**

This study reveals critical gaps in cancer awareness among university students in Bangladesh, with pronounced disparities in knowledge of nonmodifiable risk factors and significant gender-based differences in attitudes toward cancer care. These findings highlight the urgent need for targeted, gender-sensitive educational programs and policy interventions to promote preventive practices, early detection, and equitable cancer care. Such initiatives must emphasize lesser-known risk factors, reduce stigma, and foster more inclusive, culturally competent health education strategies to mitigate the growing cancer burden in Bangladesh.

## Introductions

Cancer remains one of the leading causes of morbidity and mortality worldwide, responsible for nearly 10 million deaths in 2020 alone [1]. The global burden of cancer is increasing rapidly, particularly in low- and middle-income countries, where health care infrastructure is often underdeveloped and public awareness of cancer prevention and early detection remains low [2]. Bangladesh, a densely populated low- and middle-income country, is witnessing a rising trend in cancer incidence, with an estimated 156,775 new cases and 116,598 deaths reported in 2022 [3]. These figures, however, likely underestimate the true burden due to limitations in nationwide cancer surveillance and the reliance on hospital-based cancer registries that exclude individuals without access to specialized care [4].

Common cancers in Bangladesh include lung, liver, oral cavity, and esophageal cancers in men, and breast and cervical cancers in women [5]. The underlying causes for this high prevalence are multifactorial, including environmental exposure, poor dietary habits, increasing tobacco use, and genetic predispositions, coupled with inadequate health literacy and health care access [6]. Despite ongoing efforts by governmental and nongovernmental organizations, the country continues to face significant challenges in implementing effective screening, awareness, and preventive strategies. According to World Health Organization estimates, only 5%‐10% of the population undergoes routine cancer screening, leading to late-stage diagnoses and poorer survival outcomes [7].

Awareness and attitudes toward cancer significantly shape preventive health behaviors, including risk avoidance and early detection through screening [8]. Studies globally and regionally show that young adults, especially university students, represent a critical demographic for targeted public health messaging because they are highly mobile, engaged in academic communities, and are future influencers in family and society [9,10]. However, limited studies have explored their cancer knowledge and perceptions in the Bangladeshi context.

Recent regional studies have focused primarily on breast cancer awareness among women, often neglecting broader cancer literacy and risk comprehension across genders and cancer types [11,12]. In addition, misconceptions remain widespread; many people continue to associate cancer solely with smoking or genetic predisposition while underestimating the roles of obesity, diet, reproductive factors, aging, and infectious agents in carcinogenesis [13]. Addressing these knowledge gaps is vital, as improved cancer awareness has been shown to correlate with increased participation in screening programs and healthier lifestyle choices [14].

This study aims to evaluate the awareness, knowledge, and attitudes of Bangladeshi university students toward cancer, including their understanding of risk factors, personal experiences with the disease, and attitudes toward patients and health care policies. By identifying the critical knowledge deficits and perceptual barriers in this population, this research seeks to inform culturally appropriate, gender-sensitive educational interventions to support national cancer prevention goals.

## Methods

### Participant Recruitment

Participants were recruited using a convenience sampling method through university mailing lists, social media platforms, and student networks. To minimize selection bias, efforts were made to include a diverse range of students from public and private universities located in different regions of the country.

The required sample size was calculated using Cochran’s formula for an estimated proportion of 50% (265/530), with a 95% confidence level and a 5% (27/530) margin of error, resulting in a minimum target of 385 participants. A total of 530 valid responses were collected, yielding a response rate of approximately 81.5% (432/530), after excluding incomplete or duplicate entries.

Participants were eligible if they were university students aged between 20 and 35 years. Students below or above this age range and those enrolled in primary, secondary, or higher secondary institutions were excluded.

A structured, self-administered online questionnaire was used for data collection. The questionnaire was developed based on a review of relevant literature and similar studies conducted in comparable settings. It was pretested with 30 university students to assess clarity, content validity, and internal consistency. Feedback was used to revise ambiguous questions. The final version included sections on sociodemographic characteristics, cancer knowledge, personal or family experience with cancer, and attitudes toward cancer care and policies. Cronbach α for internal reliability of the attitude-related items was .78, indicating acceptable consistency. No clinical screening or physical assessments were performed, as the study focused solely on self-reported awareness and attitudes. This study was reported in accordance with the STROBE (Strengthening the Reporting of Observational Studies in Epidemiology) statement.

### Ethical Considerations

This descriptive cross-sectional study was conducted across multiple universities in Bangladesh between December 2022 and March 2024. Ethical approval was obtained from the BRAC University Institutional Review Board (IRB No. BRACUIRB_220240006). Participation was voluntary, and informed consent was obtained before beginning the survey. Confidentiality and anonymity were strictly maintained. Data were analyzed using SPSS (version 26; IBM Corp), with categorical variables summarized as frequencies and percentages. Group differences were tested using the chi-square test, with a significance level set at *P<.05*.

## Results

Table 1 presents a detailed breakdown of the sociodemographic characteristics of the study participants. The majority of participants, 76.6% (406/530), were aged between 21 and 25 years. The gender distribution was skewed toward females, who comprised 60.4% (320/530) of the participants, compared to 39.6% (210/530) males. In terms of education levels, most participants (82.8%, 439/530) were undergraduate students, while graduate (8.5%, 45/530) and postgraduate (8.3%, 44/530) participants were nearly equal in proportion. A small fraction of participants (0.4%, 2/530) held a diploma.

**Table 1.** Sociodemographic characteristics of participants.

Characteristics	Participants (N=530), n (%)
Age (years)	
15 - 20	96 (18.1)
26 - 30	27 (5.1)
31 - 35	1 (0.2)
Sex	
Male	210 (39.6)
Female	320 (60.4)
Education	
Undergraduate	439 (82.8)
Graduate	45 (8.5)
Postgraduate	44 (8.3)
Diploma	2 (0.4)
Cancer knowledge	
Somewhat knowledgeable	322 (60.8)
Not very knowledgeable	135 (25.5)
Very knowledgeable	63 (11.9)
Not at all knowledgeable	10 (1.9)
Cancer screening	
Yes	34 (6.4)
No	496 (93.6)
You or anyone affected by cancer	
Yes	394 (74.3)
No	136 (25.7)
If yes, specify the type of cancer	
Carcinoma	280 (52.8)
Sarcoma	28 (5.3)
Leukemia	77 (14.5)
Myeloma	9 (1.7)
Lymphoma	6 (1.1)

Regarding cancer knowledge, 60.8% (322/530) of participants described themselves as “somewhat knowledgeable,” while 25.5% (135/530) reported being “not very knowledgeable.” Only 11.9% (63/530) considered themselves “very knowledgeable,” and 1.9% (10/530) indicated no knowledge of cancer. Notably, 93.6% (496/530) of participants had never undergone cancer screening. However, 74.3% (394/530) reported that they or someone they knew had been affected by cancer. Among these cases, carcinoma was the most prevalent type (52.8%, 280/530), followed by leukemia (14.5%, 77/530), sarcoma (5.3%, 28/530), myeloma (1.7%, 9/530), and lymphoma (1.1%, 6/530).

Table 2 illustrates participants’ knowledge of risk factors associated with cancer. A substantial proportion of participants identified smoking (90.9%, 482/530) and radiation exposure (86.6%, 459/530) as major risk factors. However, fewer participants recognized factors such as having more children (10.2%, 54/530) or aging (34.7%, 184/530) as risks. Family history of cancer was acknowledged by 64.2% (340/530), while only 43.2% (229/530) identified obesity as a risk factor.

**Table 2.** Impact of risk factors and symptoms knowledge of participants

Characteristics	Impact of risk factors		
	Agree, n (%)	Disagree, n (%)	Not sure, n (%)
Risk factors			
Aging	184 (34.7)	140 (26.4)	206 (38.9)
Family history	340 (64.2)	91 (17.2)	99 (18.7)
Obesity	229 (43.2)	114 (21.5)	187 (35.3)
Having more children	54 (10.2)	260 (49.1)	216 (40.8)
Radiation exposure	459 (86.6)	19 (3.6)	52 (9.8)
Infectious agents	333 (62.8)	42 (7.9)	155 (29.2)
Diet	264 (49.8)	83 (15.7)	183 (34.5)
Smoking	482 (90.9)	30 (5.7)	18 (3.4)
Artificial sweeteners	276 (52.1)	86 (12.8)	186 (35.1)

Uncertainty was notable in certain areas, with 35.1% (186/530) of participants unsure about the role of artificial sweeteners, 29.2% (155/530) uncertain about the influence of infectious agents, and 34.5% (183/530) unsure about the impact of diet. These findings highlight gaps in understanding that could be addressed through targeted educational initiatives.

Table 3 summarizes attitudes toward cancer, categorized by sex, based on various statements reflecting beliefs and perspectives on the disease. These statements address topics such as comfort levels around individuals with cancer, prioritization of patients with cancer ’ needs, government funding for cancer care and treatment, responsibility for providing care, and the necessity of accessible banking and insurance policies for patients with cancer.

The data reveal significant differences in responses between females and males for certain statements. For instance, 51.5% (273/530) of females and 33.4% (177/530) of males reported feeling comfortable around someone with cancer, with a statistically significant (*P =*.01*)*. 51.3% (272/530) of females and 33.3% (177/530) of males reported people with cancer should be given top priority, with a statistically significant (*P*=.04). Similarly, 57.2% (303/530) of females and 35.8% (190/530) of males agreed that more government funding should be allocated to cancer care and treatment (*P =.*03). Both sexes expressed support for the responsibilities toward cancer patients and the need for insurance policies, with these attitudes also showing statistical significance *(P*<.001).

Other statements explored beliefs about cancer prevention, its fatality, and its impact on careers and personal relationships. Responses to statements such as “cancer harms personal relationships,” “cancer is preventable,” “cancer means death,” and “cancer ruins careers” did not show significant differences between males and females. However, there was a significant difference in the perception of the need for increased cancer awareness, with 55.1% (292/530) of females and 35.5% (188/530) of males agreeing on its importance (*P=.*05).

**Table 3.** Gender-based differences in attitudes toward cancer care and support.

Statements and sex-wise distribution	Agree n (%)	Disagree n (%)	Not sure n (%)	*P* value
Comfortable around someone with cancer				.01
Female	273 (51.5)	29 (5.5)	18 (3.4)	
Male	177 (33.4)	30 (5.7)	3 (0.6)	
The needs of people with cancer should be given top priority				.04
Female	272 (51.3)	8 (1.5)	40 (7.5)	
Male	166 (31.3)	14 (2.6)	30 (5.7)	
More government funding should be spent on the care and treatment				.03
Female	303 (57.2)	8 (1.5)	9 (1.7)	
Male	190 (35.8)	4 (0.8)	16 (3.0)	
We have a responsibility to provide the best possible care for people with cancer				<.001
Female	273 (51.5)	41 (7.7)	6 (1.1)	
Male	181 (34.2)	16 (3.0)	13 (2.5)	
We need easy bank and insurance policy for patients with cancer				.01
Female	187 (35.3)	80 (15.1)	53 (10.0)	
Male	110 (20.8)	44 (8.3)	56 (10.6)	
Cancer is preventable				.147
Female	117 (22.1)	167 (31.5)	36 (6.8)	
Male	87 (16.4)	92 (17.4)	31 (5.8)	
Cancer means death				.996
Female	90 (17.0)	188 (35.5)	42 (7.9)	
Male	59 (11.1)	124 (23.4)	27 (5.1)	
Cancer ruin careers				0.567
Female	133 (25.1)	133 (25.1)	54 (10.2)	
Male	96 (18.1)	78 (14.7)	36 (6.8)	
Cancer harm personal relations				. 351
Female	83 (15.7)	174 (32.8)	63 (11.9)	
Male	64 (12.1)	101 (19.1)	45 (8.5)	
We need more cancer awareness				.05
Female	292 (55.1)	10 (1.9)	18 (3.4)	
Male	188 (35.5)	15 (2.8)	7 (1.3)	

## Discussion

### Principal Findings

This study assessed the knowledge and attitudes of university students in Bangladesh toward cancer, with the aim of identifying key knowledge gaps and informing targeted educational interventions. The findings revealed significant deficiencies in awareness, particularly regarding nonmodifiable risk factors such as aging and reproductive history. Gender-based differences were also prominent, with females demonstrating more supportive attitudes toward patients with cancer and policies promoting care and awareness. Although most students had some exposure to cancer through family or acquaintances, this did not translate into adequate preventive behaviors or comprehensive understanding. These results highlight an urgent need for gender-sensitive, culturally appropriate cancer education initiatives aimed at enhancing awareness, reducing stigma, and promoting early detection among young adults in Bangladesh. Research suggests that young people are a critical target audience for future cancer awareness programs [9].

The gender distribution of respondents revealed an overrepresentation of females. This aligns with previous findings suggesting that women are generally more concerned about health issues, including preventive measures, and are more likely to participate in research studies or questionnaires [10]. Conversely, studies indicate that men are less inclined to seek medical attention or adopt health-promoting behaviors, which may contribute to gender-related disparities in cancer outcomes [15]. The participants’ self-reported cancer knowledge is encouraging, with 60.8% (322/500) describing themselves as “somewhat knowledgeable” and 25.5% (135/530) considering themselves “not very knowledgeable.” However, research indicates that many young adults possess limited knowledge or awareness of cancer due to a lack of direct experience, insufficient health education, and minimal exposure to information about diseases. This aligns with the findings, which suggest a knowledge gap in this demographic [8]. A concerning aspect of the study is the low rate of cancer screening, with only 6.4% (34/530) of respondents reporting having undergone screening. Despite the relatively high self-reported awareness of cancer, the vast majority (93.6%, 496/530) had not taken any preventive screening measures. This disconnect between knowledge and action is a well-documented phenomenon in healthcare, where individuals are aware of health risks but fail to engage in preventive activities due to various barriers. These obstacles may include fear, limited access to medical services, or a perceived low personal risk [14]. For many participants, their young age may contribute to a sense of invulnerability, leading them to underestimate their risk of developing cancer despite their awareness. Additionally, 74.3% (394/530) of respondents reported that they or someone close to them had been affected by cancer, underscoring the widespread impact of the disease. Among affected individuals, carcinoma was the most prevalent type, accounting for 52.8% (280/530) of cases, followed by leukemia (14.5%, 77/530), sarcoma (5.3%, 28/530), myeloma (1.7%, 9/530), and lymphoma (1.1%, 6/530). The high prevalence of carcinoma can be attributed to its origin in epithelial cells, which are more susceptible to mutations due to frequent exposure to external carcinogens such as UV light, radiation, toxins, and chemicals. Environmental factors and lifestyle choices also contribute to the rising incidence of carcinoma in Bangladesh. The study reveals a mixed understanding of cancer risk factors among participants. Aging, a critical risk factor for cancer due to cumulative genetic mutations and reduced DNA repair capacity over time [16], was recognized by only 34.7% of participants, while 38.9% were unsure. This indicates a lack of awareness, likely stemming from the emphasis in public health campaigns on modifiable risk factors such as smoking and diet, which often overshadow non-modifiable factors like aging. Enhancing public health education to provide a balanced perspective could help underscore the significance of aging as a cancer risk factor. Family history as a risk factor was acknowledged by 64.2% (340/530) of participants, likely reflecting the success of public health messaging about genetic predispositions. However, awareness of obesity as a cancer risk factor was relatively lower, with only 43.2% (229/530) recognizing it and 35.3% (187/530) expressing uncertainty. According to the WHO, obesity’s link to cancer is often overshadowed by its associations with cardiovascular diseases and diabetes in public health discourse [13]. Regarding reproductive factors, only 10.2% of participants identified having more children as a cancer risk factor, while 49.1% (260/530) disagreed. This lack of recognition could stem from limited public health messaging on the relationship between reproductive factors and cancer risk, as well as insufficient awareness of how childbearing affects cancer risk through hormonal and genetic mechanisms [17]. Participants demonstrated high awareness of radiation exposure (86.6%, 459/530) and smoking (90.9%, 482/530) as cancer risk factors, likely due to their prominent coverage in public health campaigns and educational materials. Conversely, awareness of the role of infectious agents in cancer was comparatively lower, at 62.8% (333/530). This gap may be attributed to the complex relationship between infectious agents and cancer, which is less frequently emphasized in public health messaging. According to the WHO, the multifaceted nature of this link and its limited visibility in public discourse contribute to reduced awareness [18]. Another notable area of uncertainty was diet. While 49.8% (264/530) of participants recognized diet as a cancer risk factor, 35.1% (183/530) were unsure. Research consistently highlights that diets rich in fruits, vegetables, and whole grains are associated with a reduced risk of cancer, while diets high in processed foods and red meats are linked to increased risk [19]. Finally, 52.1% (276/530) of participants identified artificial sweeteners as a possible cancer risk factor, while 35.1% (186/530) were uncertain. The high level of uncertainty may stem from conflicting and often inconclusive scientific evidence regarding the link between artificial sweeteners and cancer risk [20]. In addition, inconsistencies in media coverage where dramatic claims are sometimes made without balanced analysis likely contribute to public confusion. Such informational disparities underscore the need for clear, evidence-based communication to reduce uncertainty in this area. The study highlights notable gender differences in attitudes toward cancer care and support in Bangladesh, revealing diverse perspectives that reflect varying levels of understanding and prioritization. Approximately 52% (273/530) of females and 34% (198/530) of males expressed positive attitudes toward patients with cancer, indicating that they would have no issues with patients with cancer living around them. This may indicate an increasing awareness among some individuals about the importance of prioritizing cancer patient needs, although other factors such as social desirability bias could also play a role. Bangladesh’s National Cancer Control Strategy and Plan of Action 2009‐2015, developed with the assistance of the WHO, aimed to establish a comprehensive continuum of cancer care [4]. However, nearly 11% (58/530) of respondents reported discomfort or inconvenience around cancer patients, likely due to limited knowledge about cancer and misconceptions regarding its transmission. A lack of understanding and awareness about cancer is further evidenced by a recent breast cancer survey in Bangladesh. According to Sarker et al, the mean knowledge score about breast cancer symptoms was 2.94 (SD 1.14) out of 8, with an overall correct response rate of only 36.8%, based on 400 participants. These findings underscore the urgent need for culturally, socially, and demographically tailored educational intervention programs to raise cancer awareness and promote self-examination practices [12]. The study also revealed that 57.2% (303/530) of females and approximately 36% (190/530) of males agreed on the importance of increased government funding for cancer treatment and care, emphasizing the need to ensure financial support for cancer patients. Financial challenges, particularly among low-income individuals, significantly impact cancer care outcomes. According to recent findings, patients with limited financial resources face severe consequences, as out-of-pocket health care expenditures in Bangladesh exacerbate financial hardship for poorer households compared to wealthier ones [6]. The higher level of agreement among women could possibly be influenced by a greater trust in governmental support systems, although this interpretation requires further investigation. However, issues of affordability remain pressing; essential services for standard breast cancer care are often inaccessible to the average Bangladeshi family due to profiteering within an overstressed healthcare system [21]. When it comes to caregiving responsibilities, 52% (273/530) of females and 34.2% (181/530) of males agreed that care for cancer patients should be provided. These differences point toward potentially complex views surrounding caregiving responsibilities; however, more in-depth qualitative research would be needed to understand the underlying beliefs. Patients who experience negative societal attitudes toward their condition are 2.5 times more likely to endure from depression than those who feel positively supported [22]. Stigmatization and societal perceptions further complicate cancer care and support for patients. The importance of accessible banking and insurance policies for cancer patients also revealed significant gender disparities, with 36% (187/530) of females and 21% (110/530) of males agreeing on their necessity. These findings underline statistically significant differences between genders across key questions related to cancer care, patient needs, and policies.

### Limitation

Ultimately, this study identified statistically significant gender differences in attitudes toward cancer care and policy; it relied primarily on bivariate analyses (eg, *χ*^2^ tests). Consequently, the observed differences may be confounded by other sociodemographic variables such as age, education level, or personal experiences with cancer. To more accurately isolate the effect of gender, future research should use multivariate analyses such as logistic regression or ANCOVA to control for potential confounders. Incorporating such methods would provide a more robust understanding of the underlying factors influencing attitudes toward cancer care.

### Conclusion

This study underscores not only a critical deficit in cancer awareness among university students in Bangladesh but also deeper societal and systemic issues that may hinder effective cancer prevention and care. The low level of screening participation, despite widespread exposure to cancer within families, reveals a significant disconnect between awareness and action reflecting broader barriers such as health system limitations, cultural attitudes, and perceived invulnerability among youth. Gender disparities in attitudes toward cancer care further suggest that public health initiatives must be responsive to sociocultural norms and gendered perceptions of health responsibility. Policies and educational programs must be redesigned to actively engage men and women in ways that acknowledge these differences, promoting inclusive, participatory cancer literacy strategies. Moreover, this research calls attention to the need for multi-level interventions that go beyond individual knowledge. Effective change will require collaboration across government, educational institutions, and health organizations to create integrated programs that normalize cancer screening, reduce stigma, and make preventive care accessible and affordable. There is also a pressing need to embed health education, particularly around non-modifiable and lesser-known risk factors, into university curricula to foster informed health behaviors early in adulthood. Finally, these findings offer a foundation for future longitudinal and qualitative studies that can explore the underlying psychological, economic, and structural barriers to cancer awareness and prevention. Addressing these broader determinants is essential for transforming knowledge into sustainable health practices and for reducing the cancer burden in Bangladesh and other low-resource settings facing similar challenges.

## Supplementary material

10.2196/75651 Checklist 1 STROBE checklist for Cross-Sectional Study.

## Abbreviations

STROBE: Strengthening the Reporting of Observational Studies in Epidemiology
WHO: World Health Organization
