# Supplementary material for: Awareness and Attitudes of University Students in Bangladesh Toward Cancer: Cross-Sectional Study
Source: JMIR Form Res. 2025 Nov 13;9:e75651. doi: 10.2196/75651 (PMC12614657; doi:10.2196/75651)
Supplement: Checklist 1 [file formative-v9-e75651-s001.docx]

STROBE Checklist for Cross-Sectional Study

Title of Manuscript: Awareness and Attitudes of University Students in Bangladesh Towards Cancer: A Cross-Sectional Study

This checklist has been completed in accordance with the STROBE guidelines for cross-sectional studies.

| Section/Topic | Item No. | Recommendation | Addressed in Manuscript |
| --- | --- | --- | --- |
| Title and Abstract | 1 | Indicate study’s design with a commonly used term; provide informative and balanced summary. | Title and Abstract (p. 1) |
| Introduction | 2 | Background/rationale—Explain scientific background and rationale. | Introduction (p. 2–3) |
|  | 3 | Objectives—State specific objectives, including prespecified hypotheses. | Introduction (end, p. 3) |
| Methods | 4 | Study design—Present key elements of design early in the paper. | Methods (p. 3) |
|  | 5 | Setting—Describe setting, locations, and dates, including periods of recruitment and data collection. | Methods, Recruitment (p. 3) |
|  | 6a | Participants—Eligibility criteria, sources, and methods of selection. | Methods (p. 3–4) |
|  | 7 | Variables—Clearly define outcomes, exposures, predictors, and confounders. | Methods (p. 4) |
|  | 8 | Data sources/measurement—Sources of data and methods of assessment. | Methods (p. 4) |
|  | 9 | Bias—Describe efforts to address bias. | Methods, Recruitment (p. 3) |
|  | 10 | Study size—Explain how the study size was arrived at. | Methods, Sample size (p. 3) |
|  | 11 | Quantitative variables—Explain handling of quantitative variables in the analysis. | Methods (p. 4) |
|  | 12a | Statistical methods—Describe all statistical methods including for subgroup analyses. | Methods (p. 4) |
|  | 12b–d | Describe any methods to examine subgroups/interactions, address missing data, and conduct sensitivity analysis. | Limited (no missing data strategy stated; no sensitivity analysis done) |
| Results | 13a | Participants—Report numbers at each stage of the study. | Results, Table 1 (p. 5) |
|  | 13b | Give reasons for non-participation. | Not detailed |
|  | 14a | Descriptive data—Provide characteristics of participants. | Results, Table 1 (p. 5) |
|  | 14b | Indicate number of participants with missing data. | Not specified |
|  | 15 | Outcome data—Report outcomes or summary measures. | Results (p. 5–7, Tables/Fig.) |
|  | 16a | Main results—Unadjusted estimates and precision (e.g., confidence intervals). | Chi-square results (p. 7) |
|  | 16b–c | Report confounder-adjusted estimates and discuss categorical boundaries. | Not done (no multivariate analysis) |
| Discussion | 18 | Key results—Summarize key findings in relation to study objectives. | Discussion (p. 8–10) |
|  | 19 | Limitations—Discuss limitations of the study. | Discussion, briefly noted |
|  | 20 | Interpretation—Give cautious overall interpretation considering other studies. | Discussion (p. 8–10) |
|  | 21 | Generalizability—Discuss external validity of results. | Discussion (end of p. 10) |
| Other information | 22 | Funding—State source of funding and role of funders. | Acknowledgment (p. 11) |
